# Impact of mHealth on Medication Adherence in Older Adults with Chronic Diseases Facing Treatment Burden: A Systematic Review

**DOI:** 10.3390/geriatrics11040078

**Published:** 2026-07-02

**Authors:** Leandro Amato, Isabella Napoleoni, Noemi Giannetta, Emanuele Di Simone, Nicolò Panattoni, Alessandra Improta, Erika Renzi, Azzurra Massimi, Marco Di Muzio, Sofia Taborri

**Affiliations:** 1 Department of Biomedicine and Prevention, University of Rome Tor Vergata, 00133 Rome, Italy; leandro.amato@uniroma1.it (L.A.); sofia.taborri@uniroma1.it (S.T.); 2 Department of Clinical and Molecular Medicine, Sapienza University of Rome, 00185 Rome, Italy; isabella.napoleoni85@gmail.com; 3 Departmental Faculty of Medicine, Saint Camillus International University of Health and Medical Sciences (UniCamillus), 00131 Rome, Italy; noemi.giannetta@unicamillus.org; 4 Department of Medical, Movement and Wellbeing Sciences, Parthenope University of Naples, 80133 Naples, Italy; emanuele.disimone@uniparthenope.it; 5 Nursing Research Unit IFO, IRCCS Regina Elena National Cancer Institute, 00144 Rome, Italy; nicolo.panattoni@ifo.it; 6 Department of Public Health and Infectious Diseases, Sapienza University of Rome, 00185 Rome, Italy; erika.renzi@uniroma1.it (E.R.); azzurra.massimi@uniroma1.it (A.M.); 7 Department of Wellbeing, Health and Environmental Sustainability, Sapienza University of Rome, 00185 Rome, Italy; marco.dimuzio@uniroma1.it

**Keywords:** older adult, mHealth, mobile app, medication adherence, chronic disease

## Abstract

**Background**: In older adults, multimorbidity and polypharmacy complicate medication regimens and often lead to poor adherence. Mobile health (mHealth) has been suggested as a solution to enhance medication adherence in chronic conditions. Despite the increase in smartphone usage among people aged 65 and over, there is still a lack of evidence of mHealth in this age group. **Objectives**: To evaluate the impact of mHealth interventions on medication adherence in older adults (≥65 years) with chronic diseases, compared with standard care or other interventions. **Methods**: The review was conducted in accordance with PRISMA 2020 guidelines and the Cochrane Handbook for Systematic Reviews. Randomized controlled trials published from 2000 onwards were considered with no linguistic or geographical restrictions. The databases searched included PubMed, Scopus, Cochrane Library, and CINAHL. Methodological quality was assessed using the Revised Cochrane Risk of Bias Tool for Randomized Trials. **Results**: 551 records were initially identified, from which 8 randomized controlled trials published between 2014 and 2025 were included. Six of eight studies showed that medication adherence in mHealth groups was significantly higher than in controls. However, one study found benefits only in specific drug classes rather than a general improvement. **Conclusions**: The results of this review suggest that mHealth has the potential to improve medication adherence among older adults with chronic diseases, especially when interventions go beyond simple reminders and incorporate educational and relational components. Nevertheless, higher quality studies with larger samples and longer follow-up are needed to clarify mHealth’s role in the care of this population.

## 1. Introduction

Population ageing represents a major challenge for healthcare systems worldwide. According to the World Health Organization, people aged 65 or over are considered ‘elderly’ [1]. By the late 2070s, the global population aged ≥65 years is projected to reach approximately 2.2 billion, outnumbering those younger than 18 [2]. This shift comes amid an epidemiological transition: acute and infectious diseases decline, while chronic conditions now cause 75% of global deaths [3,4].

In older adults, the frequent coexistence of multiple chronic conditions often leads to complex therapeutic regimens involving the simultaneous prescription of five or more medications, a condition known as polypharmacy [5]. The World Health Organization defines medication adherence as the degree to which a person’s behavior matches agreed medical advice [6]. Both multimorbidity and polypharmacy challenge adherence by increasing treatment burden, the risk of adverse effects and the complexity of self-management, all of which may undermine a patient’s ability to follow prescribed regimens consistently [5,6].

Poor medication adherence is highly prevalent among older patients [7], with estimated rates ranging from 21% to 55% [8]. Non-adherence results in treatment failure, disease progression, increased hospitalizations and healthcare costs, and higher mortality rates [9]. Treatment effectiveness cannot be achieved without improving medication adherence, which requires targeted strategies, especially for older people. This issue is largely attributable to the presence of multiple age-related factors that directly influence adherence to prescribed therapies. These factors include impaired neurocognitive and psychological functioning, sensory deficits, frailty, the burden of chronic medical conditions, physical limitations, inadequate health literacy, and patients’ beliefs and perceptions regarding medications, healthcare providers, and the healthcare system itself [10]. Digital health literacy is also relevant in this population, as older adults with higher digital competencies tend to make more effective use of technology-based tools [11]. Mobile health (mHealth) has been proposed to support chronic disease management and medication adherence. mHealth uses mobile devices to support medicine and public health, facilitating prompt healthcare delivery, enhancing communication between providers and patients, and encouraging patient participation [12,13]. Its key features include reminders, monitoring, educational resources and direct communication between patients and healthcare professionals [14]. Smartphone usage is increasing among older adults globally; in the United States, for example, 61% of those aged 65 and older had a smartphone in 2022 [15]. Through these devices, older adults can access information on disease prevention, rehabilitation, and medication management [16,17]. In the geriatric context, the evaluation of mHealth interventions should go beyond clinical efficacy to consider acceptability and usability as equally important outcomes [18].

Despite growing interest in digital health strategies, previous reviews on mHealth have either examined outcomes other than medication adherence or focused predominantly on adult populations without specifically targeting older adults [14,19,20,21,22]. Given the progressive ageing of the global population, the increasing prevalence of chronic diseases, and the spread of digital technologies that may effectively support adherence in this group, there is a clear need to address the existing knowledge gap.

In this systematic review, we aimed to assess the impact of mHealth interventions designed to support medication adherence among older adults with chronic diseases, a population often managing multiple conditions, polypharmacy, and high treatment burden, compared with standard care or other interventions. The goal is to provide a complete and updated summary of the available scientific evidence, along with insights and directions for future research in order to develop more tailored strategies that address poor medication adherence and meet the care needs of a rapidly aging global population.

## 2. Materials and Methods

This systematic review was conducted in accordance with the recommendations of the Cochrane Handbook for Systematic Reviews of Interventions (version 6.5) [23] and reported following the PRISMA 2020 (Preferred Reporting Items for Systematic Reviews and Meta-Analyses) guidelines [24]; the PRISMA checklist is provided as Supplementary File S1. This systematic review was registered on PROSPERO (registration number CRD420251246319). The methodological quality of the included studies was assessed using the Revised Cochrane Risk of Bias Tool for Randomized Trials (RoB 2) [25]. These measures, along with independent screening and data extraction by two reviewers, were adopted to minimize bias in study selection and reporting.

### 2.1. Search Strategy

A comprehensive literature search was conducted between December 2024 and April 2026 across PubMed, Scopus, CINAHL, and the Cochrane Library. The search strategy was developed based on the review question formulated according to the PICO framework (Population, Intervention, Comparison, Outcome) [26]: “What are the effects of mHealth on medication adherence in older adults with chronic diseases compared with standard care or other interventions?”.

PICO-based keywords were selected and combined with Boolean operators to create a search string that was tailored to each database (see Supplementary File S2). Search terms included older adult, aged patient, chronic disease, mobile applications, mHealth, medication adherence, and treatment compliance. Studies were included if participants were at least 65 years old, either by minimum age or sample mean. This criterion was used to expand the inclusion of studies involving older adults, due to the limited number of studies conducted exclusively on populations aged 65 and above.

Participants were required to have at least one chronic condition and to be community-dwelling (as institutionalized patients are not primarily responsible for their own medication self-management). We excluded studies with participants under 65 years old, healthy individuals, participants with acute conditions, or institutionalized patients.

Eligible studies used mobile health interventions aimed at improving medication adherence. All technologies delivered through mobile or portable devices, such as smartphone and tablet apps and video conferencing platforms, were considered. Interventions could be used alone or combined with other interventions or devices. Studies lacking mobile-based interventions were excluded. Medication adherence was required to be evaluated as a primary or secondary outcome.

Only randomized controlled trials (RCTs) with full-text availability were included in the review.

The search was limited to articles published from 2000 onwards, marking the period when the first developments and applications of mobile health began to emerge [27]. No restrictions on language, geographical origin, or gender were applied. We excluded grey literature, qualitative studies, editorials, surveys, opinions, feasibility studies, protocols, and secondary studies.

### 2.2. Study Selection

Records retrieved from the database searches were aggregated and managed using Zotero software (version 7.0.22; Corporation for Digital Scholarship, Vienna, VA, USA) [28].

The screening process was carried out in three stages. Initially, the titles of all identified records were reviewed, and duplicate studies were removed. Abstracts were then screened, and the full texts of potentially relevant studies were retrieved, excluding those that did not satisfy the inclusion criteria.

Finally, full-text articles were examined in detail, and only studies meeting all eligibility criteria were included in the review. The entire selection process was documented and reported using a PRISMA flow diagram.

Screening was performed independently by two reviewers. Any discrepancies were resolved through discussion between the reviewers or, when necessary, by consultation with a third reviewer.

### 2.3. Data Extraction and Synthesis

Data were extracted independently by two reviewers from the included studies using a summary table in Microsoft Excel (Microsoft Corporation, Redmond, WA, USA). The following data were extracted from the included studies: author(s) and year of publication, sample size, mean age and clinical characteristics of participants, follow-up duration, type of mHealth device and related functionalities, and main adherence outcomes. A narrative synthesis of the selected studies was subsequently performed to identify the most consistent findings and to highlight the main differences among studies. Due to the clinical and methodological heterogeneity of the included studies, as well as the limited number of available studies, a meta-analysis was not conducted.

## 3. Results

### 3.1. Search Results

The literature search conducted across the four databases initially identified 551 records. After removing 178 duplicates and excluding an additional 170 records based on title screening, abstracts were assessed, resulting in the exclusion of 52 studies.

A total of 151 full-text articles were sought for retrieval; however, 35 could not be obtained, resulting in 116 articles assessed for eligibility.

**Figure 1:**
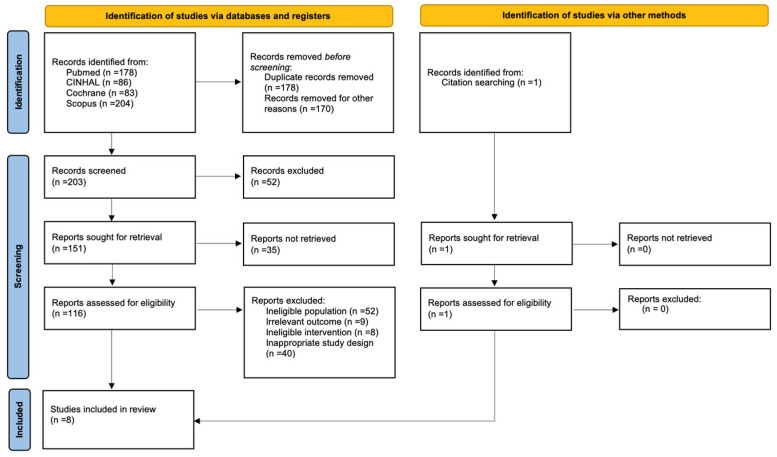
PRISMA flowchart.

At this stage, 109 articles were excluded. The main reasons for exclusion included ineligibility of the study population, the intervention, the outcome assessed, or the study design. An additional study was identified through the reference lists of included papers (backward citation searching). At the end of the screening process, 8 studies were included in the systematic review. The entire identification and selection process is illustrated in the PRISMA flow diagram below (Figure 1).

### 3.2. Characteristics of Studies

All included studies were RCTs, including two cluster RCTs [29,30] and one crossover design [31]. The included studies were published between 2014 and 2025 and showed heterogeneous follow-up durations, ranging from 56 days to 12 months. Sample sizes ranged from 24 [31] to 1299 older adults [30]. Two studies were conducted in China [29,30], while the remaining studies were carried out in India [32], Spain [33], Germany [31], South Korea [34], the United States [35], and Iran [36]. The study populations consisted of older adults, with a mean age ranging from 65.7 years [30] to 73.8 years [31], all affected by at least one chronic condition.

Cardiovascular diseases were the most frequently represented clinical conditions, including stroke [30], hypertension [29], heart failure [35], and ischemic heart disease [31]. In four studies, participants were described as having one or more chronic conditions without further specification [32,33,34,36].

The risk of bias was evaluated using the Revised Cochrane Risk-of-Bias Tool for Randomized Trials, since all included studies adopted a randomized controlled trial design. Overall, none of the eight included studies was judged to be at high risk of bias, although each study presented at least one domain classified as “some concerns”.

Domain 2 (risk of bias due to deviations from intended interventions) was the most frequently affected, with all studies rated as “some concerns”. Domain 4 (bias in outcome measurement) also emerged as a critical area; only Yan et al. [30], Poorcheraghi et al. [36], and Hwang et al. [34] were judged to be at low risk of bias in this domain. A detailed summary of the risk-of-bias assessment is provided in the Supplementary Material (see Supplementary File S3).

### 3.3. Characteristics of mHealth Interventions

As shown in Table 1, the included interventions were classified according to four functional components: medication reminders (R), educational content (E), monitoring of clinical parameters (M), and communication with healthcare professionals or caregivers (C). The analysis of the included studies highlights the heterogeneity of mobile health solutions, both in terms of device type and the functionalities they offer. The identified devices were divided into three main groups: mobile phones, tablets, and other devices.

In five of the included studies, interventions were based on mobile phone–based solutions [29,30,32,34,36]. In two of these studies, the intervention involved dedicated applications providing medication reminders, access to patient educational content, communication with healthcare professionals or caregivers, and monitoring of symptoms or clinical parameters, delivered via mobile phone alone [36] or in combination with a smart band [34]. In one study, the intervention relied on automated text messaging, used to provide medication reminders, convey educational content, and facilitate communication with healthcare professionals or caregivers [29]. In two studies, integrating voice-based components, such as pre-recorded voice messages or scheduled phone calls, enabled the medication reminder to be linked to educational information and to a direct connection with healthcare providers or caregivers [30,32]. One of these studies also included monitoring of symptoms or clinical parameters [30].

The second category of interventions involved tablet-based applications, described in two studies [31,33]. In one study, the device combined medication reminders with educational content [33], while in the other, reminders were integrated with a function for monitoring symptoms or clinical parameters [31].

Finally, the third category included a study involving a multicomponent mobile device connected to a remote monitoring center, integrating medication reminders, remote monitoring of vital parameters, and communication with healthcare professionals or caregivers [35].

### 3.4. Impact of mHealth on Medication Adherence

In six out of eight studies, a statistically significant improvement in medication adherence was observed in the intervention groups compared with control groups [29,30,31,32,33,36], although one of these [30] showed benefits limited to specific drug classes rather than a generalized improvement. The remaining two studies did not report significant differences between groups [34,35]. Regarding mobile phone–based interventions, results were heterogeneous. In one study, the use of a dedicated mobile application led to a significant improvement in medication adherence, measured through a self-report scale, compared with the control group (*p* < 0.001) [36]. Hwang et al. [34] did not find statistically significant differences in adherence between groups (*p* = 0.138).

All three studies based on SMS systems and automated phone calls showed a significant improvement in medication adherence compared with control groups. In particular, Raj et al. [32] reported significantly higher medication adherence in the intervention group at three months (*p* = 0.007) and six months (*p* = 0.003), measured by pill count. Similarly, Zhai et al. [29] showed that the intervention group had a significantly greater level of adherence, measured by self-assessment (*p* = 0.04), than the control group. Yan et al. [30] found that the intervention group had significantly improved adherence to statins (*p* = 0.003) and antihypertensive drugs (*p* = 0.039), measured by self-report; however, neither intervention had a statistically significant effect on antiplatelet agents (*p* = 0.658). Both studies that evaluated the tablet-based application reported that the intervention group had significantly higher medication adherence than the control group. Mira et al. [33] reported that the intervention group had a statistically significant increase in self-reported adherence scores compared to those receiving standard care (*p* < 0.001). Mertens et al. [31] compared the tablet application to a paper diary instead of standard care and found that the tablet application resulted in significantly higher self-reported adherence scores (*p* = 0.02). Notably, both groups showed a significant improvement from baseline (*p* < 0.001), suggesting that structured monitoring itself, whether delivered digitally or through paper-based tools, may positively influence medication adherence.

Finally, the study by Hale et al. [35], which evaluated a multicomponent mobile device, did not report statistically significant differences in medication adherence when compared with the control group (*p* = 0.61).

### 3.5. Measurement of Medication Adherence

The present review shows that the most widely used tools for assessing medication adherence were self-report scales, which allow patients to directly report their medication-taking behaviors. In two studies, the 4-item version of the Morisky Medication Adherence Scale (MMAS-4) [37] was used [30,33], while three studies [29,34,36] adopted the 8-item version (MMAS-8) [38]. In one study [31], adherence was assessed using the A14-scale [39], and in another [35], the Medical Outcomes Study single-item adherence question (MOS) was used [40]. Pill count was used in two of eight studies [32,36], whereas in three studies, adherence was measured using digital intake confirmation logs and data from electronic devices, integrated with self-report scales [31,33,35]. Table 2 summarizes the medication adherence assessment tools used in the included studies.

## 4. Discussion

The present review aimed to evaluate the impact of mobile health interventions on medication adherence among older adults with chronic diseases, compared with standard care or other interventions. In six of the eight included studies, the use of mobile devices, particularly mobile phones [29,30,32,36] and tablets [31,33], was associated with significantly higher medication adherence compared with control groups.

These findings align with previous systematic reviews conducted in a broader adult population, which consistently report improvements in adherence to drug therapy through mHealth interventions across several chronic conditions [14,41,42,43,44,45]. This review extends this evidence to older adults as a distinct population warranting targeted investigation. Beyond statistical significance, these findings may also have relevant clinical implications. In older adults with chronic diseases, even modest improvements in medication adherence may contribute to reducing missed doses, improving continuity of treatment, and enhancing disease control [6,10]. This aspect is particularly important in the presence of multimorbidity and polypharmacy, where poor adherence is associated with disease progression, avoidable hospitalizations, and increased healthcare utilization [9]. Nevertheless, the clinical significance of the available evidence should be read carefully. In fact, most of the included studies evaluated medication adherence as the primary outcome and were not designed to assess whether improved adherence translates into better clinical outcomes, such as improved disease control, reduced complications, or lower hospitalization rates [46].

Available mHealth interventions for supporting medication adherence range from low-complexity solutions, such as SMS and phone calls, to more advanced systems, including dedicated smartphone and tablet applications, multicomponent mobile devices, and integrated platforms. In the studies analyzed, the most commonly adopted interventions were those based on mobile phone use [29,30,32,34,36], possibly reflecting the increasing availability of smartphones in this population. Data from the United States indicate that smartphone ownership among individuals aged 65 and older reached 61% in 2022 [15], suggesting a growing technological readiness in this age group, although penetration rates vary across countries and settings. Consistent with existing literature, favorable results were also observed in studies using tablets as a therapeutic management support tool [31,33].

The interventions that showed the most consistent results were those that integrated the reminder function for drug intake with interactions with healthcare professionals [29,30,32,36]. This finding aligns with previous evidence. Reminder systems address a concrete need among older adults: remembering to take medications and avoiding omissions or duplications, which are common in patients undergoing polypharmacy [47,48,49]. However, several studies suggest that reminders used as standalone interventions, without integration into more structured strategies, do not consistently result in significant improvements in clinical outcomes [50,51]. For this reason, the most effective mHealth interventions tend to integrate educational and motivational components, which are recognized as crucial for promoting medication adherence among patients with chronic diseases [46,52].

The interaction between healthcare professionals and patients represents an additional factor that may enhance medication adherence in older adults. Numerous studies have shown that effective communication with healthcare providers promotes healthy behaviors and greater engagement in care pathways [53]. In particular, direct contact with professionals enables the provision of clear and reassuring information, reducing doubts and uncertainties [54]. This aspect is especially relevant in older populations, who may experience difficulties in correctly interpreting prescriptions, recognizing warning signs, or managing potential side effects, especially in the presence of multimorbidity and polypharmacy [55]. Studies that incorporated direct communication between patients and healthcare professionals or caregivers, through scheduled phone calls [32] or app-mediated messaging [29,30,36], tended to show stronger adherence outcomes. These findings suggest that mHealth interventions may be most effective when designed not only as reminder tools, but as platforms that strengthen the relational dimension of care [29,30,32,36].

While the widespread use of and familiarity with devices such as smartphones and tablets appear to contribute to the effectiveness of interventions based on these technologies, interventions that are less integrated into older adults’ daily routines may be less effective. An example is the study by Hale et al. [35], which found that using the multicomponent mobile device MedSentry did not result in statistically significant differences in medication adherence compared with the control group. Similarly, Hwang et al. [34], who implemented a mobile health intervention combining a mobile phone and a smart band, also reported non-statistically significant differences in medication adherence scores compared with usual care [34]. This finding is consistent with the literature, which suggests that the effectiveness of digital health interventions depends not only on their technological features but also on usability, integration into clinical practice, and user acceptability [56,57]. Additionally, the study by Hale et al. [35] included a relatively small sample (*n* = 25), which may have limited its statistical power; therefore, the lack of significant findings may reflect underpowering rather than true ineffectiveness of the intervention.

A particular case is the study by Yan et al. [30], which found that the mHealth intervention improved medication adherence compared with standard care but was limited to certain drug classes. This variability has been attributed in the literature to several factors, including the occurrence of side effects, perceived limited treatment effectiveness, frequent physician-initiated therapy modifications, and healthcare system-related constraints, such as costs or restricted access to medications [58,59].

These findings, however, may not apply equally to all older adults. Frail individuals, those with cognitive impairment, and those with limited digital access may face additional barriers to adopting and using mHealth tools [60,61], and the benefits observed in controlled trial settings may not hold in these groups.

Future studies should investigate large-scale randomized controlled trials with adequate sample sizes and long-term follow-up to measure mHealth’s effect on the long-term sustainability of medication adherence. Outcomes beyond adherence, such as hospitalization rates, disease control, quality of life, and treatment burden, should also be considered, to assess whether improvements in adherence translate into measurable clinical benefits. Electronic monitoring devices, such as the Medication Event Monitoring System (MEMS), record the date and time of each medication container opening, allowing a more objective and continuous tracking of adherence over time [62]. Future trials should consider incorporating them. When comparing interventions based only on reminders with those that also include educational, motivational, and relational components, future studies could establish the specific contribution of each intervention to the intervention effectiveness. Furthermore, future research should explore the potential of mHealth devices not only as interventions but also as tools for continuous data collection on patient behavior over time, providing a more complete picture of how adherence evolves in older adults with chronic diseases [63,64].

In addition, using standardized tools to measure adherence would enhance consistency across studies and facilitate future meta-analysis.

As the present review focused on the effectiveness of mHealth interventions assessed through randomized controlled trials, feasibility studies were not included. Future research should explore acceptability, usability, and implementation barriers to better understand the applicability of mHealth interventions in older adults.

Finally, given that most existing evidence derives from high-income settings, future studies should specifically target older adults in low- and middle-income countries, where access to technology and healthcare infrastructure differ substantially.

### Limitations

Although this review employed a rigorous methodological approach in line with established guidelines, several limitations should be considered when interpreting the findings. First, the limited number of studies, small sample sizes, and/or limited follow-up duration in several of the included studies (i.e., many studies focused on short- or medium-term outcomes) led to a lack of information on the long-term effects of mHealth interventions. In addition, few total studies focused on this population, which was an additional limitation. The decision to include only studies published from 2000 onwards and to limit the analysis to RCTs, while enhancing the methodological rigor of the review, may have led to the exclusion of potentially relevant prior contributions. Similarly, the exclusion of grey literature may have resulted in the loss of useful information. Furthermore, since eligibility was based on mean age, there is a risk that some included samples comprised participants younger than 65 years of age. Some studies enrolled adults aged ≥60 years [31,32,36] or did not specify a lower age cutoff [29,35], introducing a substantial risk of misclassification. As a result, the findings may not be fully representative of the older adult population. The included studies showed significant differences in geographical distribution, study settings, and the variety of mHealth interventions evaluated. The interventions evaluated also varied by type, intensity, and duration, as well as the presence of complementary components, making it difficult to determine how much the mHealth intervention contributed to an improvement in medication adherence during the evaluation. The methodological heterogeneity of studies that were included in this review was further increased by differences in study design. As a meta-analysis was not conducted, no pooled effect size or formal measure of statistical heterogeneity was calculated.

Two studies [29,30] adopted a cluster-randomized controlled trial, while one study [31] used a crossover design. This may limit comparability with studies that used individual randomization, given the different methodological approaches across the studies reviewed. Additionally, the studies used different methods to assess therapeutic adherence, further contributing to heterogeneity among the studies reviewed. Consequently, the finding that six out of eight studies reported statistically significant improvements in adherence must be read in light of this heterogeneity, as statistical significance does not in itself indicate clinical relevance. Moreover, self-report scales and pill count, the most used tools across the included studies, are weak measures of actual medication intake, as they are prone to social desirability and recall bias and tend to overestimate adherence, potentially inflating the observed intervention effects. The risk-of-bias assessment indicated methodological concerns across all included studies and a need for future research with more rigorous designs.

Finally, a potential publication bias cannot be excluded, as six out of eight included studies reported positive findings, suggesting that negative or non-significant results may be underrepresented in the literature.

## 5. Conclusions

The findings of this review suggest that mHealth interventions represent a promising strategy to improve medication adherence among older adults with chronic diseases. The majority of the included studies reported improvements compared with standard care, especially when interventions extended beyond simple medication reminders and included educational components, motivational elements, and interactions with healthcare professionals. However, given the heterogeneity of the adherence measurement tools used across studies, whether these results translate into clinically meaningful benefits remains unclear. Smartphones and tablets seem particularly well suited for older adults, as these devices are often already part of patients’ everyday lives and are easy to use. These factors may contribute to their effectiveness in supporting medication adherence.

These findings may have important implications for clinical practice. Current evidence suggests that mHealth interventions could represent a useful complementary approach for supporting medication adherence among older adults with chronic conditions. For this reason, healthcare professionals may consider integrating such tools when planning or recommending adherence-support strategies for this population.

However, these results must be read carefully. The current evidence is limited by the small number of studies, small sample sizes, short follow-up periods, heterogeneous interventions, and frequent reliance on self-reported measures, and this should be kept in mind when interpreting the findings. As a result, stronger evidence from large-scale, methodologically rigorous studies with standardized outcome measures and longer follow-up periods is still needed before firm recommendations for clinical practice and health policy can be established.

## Figures and Tables

**Table 1:** Characteristics of the included studies.

Authors (Year)	Sample (*n*; Age ± SD, yrs)	Condition	Follow-Up	mHealth Device	mHealth Functionalities	Adherence Outcomes
Mira et al. (2014) [33]	*n* = 99 70.9 ± 8.0	Unspecified multimorbidity	3 months	Tablet	R + E	+
Mertens et al. (2016) [31]	*n* = 24 73.8 ± 7.5	Ischemic heart disease/recent MI	56 days	Tablet	R + M	+
Hale et al. (2016) [35]	*n* = 25 71.7 ± 11.2	Heart failure	3 months	Multicomponent mobile device	R + M + C	−
Raj et al. (2020) [32]	*n* = 50 69.1 ± 7.1	Unspecified multimorbidity	6 months	Mobile phone	R + E + C	+
Zhai et al. (2020) [29]	*n* = 384 68.5 ± 7.9	Hypertension	3 months	Mobile phone	R + E + C	+
Yan et al. (2021) [30]	*n* = 1299 65.7 ± 8.2	Stroke	12 months	Mobile phone	R + M + E + C	+ (statins, antihypertensives) − (antiplatelets)
Poorcheraghi et al. (2023) [36]	*n* = 184 68.9 ± 5.2	Unspecified multimorbidity	2 months	Mobile phone	R + M + E + C	+
Hwang et al. (2025) [34]	*n* = 42 72.9 ± 3.4	Unspecified multimorbidity	2 months	Mobile phone + smart band	R + M + E + C	−

SD: standard deviation; MI: myocardial infarction; R: medication reminders; E: educational component; M: monitoring of clinical parameters/symptoms; C: communication with healthcare professionals/caregivers; (+): statistically significant improvement in medication adherence; (−): no significant difference between groups. All studies used usual care as the control condition, except Mertens et al. [31], in which the comparator was a paper diary.

**Table 2:** Medication adherence assessment tools.

Study	Measurement Tool	Characteristics
Poorcheraghi et al. (2023) [36]	MMAS-8 + pill count	8-item self-report questionnaire (score 0–8), classifying adherence as low, medium, or high + pill count based on the number of remaining tablets compared with those prescribed
Hale et al. (2016) [35]	MOS questionnaire + MedSentry device data	Single-item self-report questionnaire (adherent/non-adherent) + automatic device log
Raj et al. (2020) [32]	Reported Pill Count	Count of remaining tablets compared with those prescribed; result expressed as percentage of doses taken
Mira et al. (2014) [33]	MMAS-4 + ALICE app data	4-item yes/no self-report questionnaire (score 0–4: high, medium, low adherence) + automatic device log
Mertens et al. (2016) [31]	A14-scale + Medication Plan app data	14-item self-report questionnaire (score 0–56; <50 = non-adherent, ≥50 = adherent) + automatic device log
Zhai et al. (2020) [29]	MMAS-8	Validated 8-item self-report questionnaire (score 0–8: low <6, medium 6–<8, high = 8)
Yan et al. (2021) [30]	MMAS-4	4-item yes/no self-report questionnaire; score 0 = adherent, ≥1 = non-adherent
Hwang et al. (2025) [34]	MMAS-8	Validated 8-item self-report questionnaire (score 0–8: low <6, medium 6–<8, high = 8)

## Data Availability

No new data were generated.

## Supplementary Materials

The following supporting information can be downloaded at: https://www.mdpi.com/article/10.3390/geriatrics11040078/s1. File S1: PRISMA 2020 Checklist; File S2: Search Strategy; File S3: Risk of Bias Assessment.

## Abbreviations

The following abbreviations are used in this manuscript:

CINAHL	Cumulative Index to Nursing and Allied Health Literature
mHealth	Mobile Health
MMAS-4	Morisky Medication Adherence Scale, 4 items
MMAS-8	Morisky Medication Adherence Scale, 8 items
MOS	Medical Outcomes Study
PICO	Population, Intervention, Comparison, Outcome
PRISMA	Preferred Reporting Items for Systematic Reviews and Meta-Analyses
RCT	Randomized Controlled Trial
RoB 2	Revised Cochrane Risk of Bias Tool for Randomized Trials
SD	Standard Deviation
SMS	Short Message Service
